# Clinical significance of soluble programmed cell death-1 and soluble programmed cell death-ligand 1 in patients with locally advanced rectal cancer treated with neoadjuvant chemoradiotherapy

**DOI:** 10.1371/journal.pone.0212978

**Published:** 2019-02-26

**Authors:** Tetsuro Tominaga, Takashi Akiyoshi, Noriko Yamamoto, Senzo Taguchi, Seiichi Mori, Toshiya Nagasaki, Yosuke Fukunaga, Masashi Ueno

**Affiliations:** 1 Gastroenterological Center, Department of Gastroenterological Surgery, Cancer Institute Hospital, Japanese Foundation for Cancer Research, Koto-ku, Tokyo, Japan; 2 Division of Pathology, Cancer Institute, Japanese Foundation for Cancer Research, Tokyo, Japan; 3 Department of Radiation Oncology, Cancer Institute Hospital, Japanese Foundation for Cancer Research, Tokyo, Japan; 4 Cancer Precision Medicine Center, Japanese Foundation for Cancer Research, Tokyo, Japan; University of South Alabama Mitchell Cancer Institute, UNITED STATES

## Abstract

**Background:**

Inhibition of the programmed cell death-1/programmed cell death-ligand 1 (PD-1/PD-L1) axis in combination with radiotherapy may be a promising approach to treat cancer. In the present study, we aimed to evaluate serum soluble PD-1/PD-L1 levels in patients with advanced rectal cancer treated with neoadjuvant chemoradiotherapy (CRT).

**Methods:**

Serum soluble PD-L1 and PD-1 levels were measured using an enzyme-linked immunosorbent assay before and after CRT in 117 patients with low rectal cancer. Changes in the levels of sPD-L1/PD-1 after CRT, and the correlation between sPD-L1/PD-1 level and clinicopathological characteristics or disease-free survival (DFS) were evaluated.

**Results:**

sPD-L1 levels significantly increased after CRT (p < 0.0001), whereas sPD-1 levels did not change significantly (p = 0.1050). High sPD-L1 before CRT was significantly associated with younger age (p = 0.044), and after CRT, with lymphovascular invasion (p = 0.021). High sPD-1 before and after CRT was significantly associated with a longer distance of the tumor from the anal verge (both p < 0.001). There was no correlation between sPD-L1 level and local PD-L1 expression on stromal immune cells. High sPD-L1 level after CRT tended to be associated with worse DFS (p = 0.0752). The multivariate analysis could not demonstrate an independent association for sPD-L1 levels after CRT with DFS.

**Conclusions:**

Significant increase of sPD-L1 levels after CRT suggests that anti-PD-L1 therapy might be a potential treatment strategy in combination with CRT in advanced rectal cancer.

## Introduction

Neoadjuvant chemoradiotherapy (CRT) is the standard of care for locally advanced rectal cancer. Several randomized, controlled trials (RCTs) have demonstrated that neoadjuvant CRT reduces the local recurrence rate in these patients [1–4]. However, CRT has no significant effect on long-term outcome, and distant metastasis is still the dominant cause of death after CRT. Alternative strategies incorporating systemic chemotherapy before or just following neoadjuvant CRT have been investigated in some clinical trials, but there is insufficient evidence to show that such strategies reduce metastatic risk as compared with conventional CRT [5, 6]. Therefore, novel treatment strategies are necessary to improve the oncological outcomes in patients with advanced rectal cancer.

Programmed cell death-1 (PD-1) is an immunoglobulin superfamily transmembrane protein mainly expressed on T cells. Programmed cell death-ligand 1 (PD-L1) is one of the ligands of PD-1[7] expressed on the surface of activated T cells and macrophage lineage cells or aberrantly expressed on tumors. Binding of PD-L1 to PD-1 inhibits T-cell activation, leading to immune suppression. Antibodies against PD-1 and PD-L1 can improve the clinical outcomes of patients with several different tumor types [8].

PD-L1 and PD-1 exist as membrane-bound and soluble forms [7, 9]. Soluble PD-L1 (sPD-L1) is released from PD-L1-positive cells, binds to the receptor of PD-1, and causes immune suppression and immune damage [10, 11]. sPD-L1 levels are increased in patients with some malignancies, and are closely correlated with poor oncological outcomes [7, 9]. Among patients with mismatch-repair proficient colorectal cancer, tumoral PD-L1 expression levels are very low (<3%) [12, 13], and immune checkpoint inhibitors alone are not effective [14]. However, some studies have shown elevated PD-L1 expression in rectal cancer after CRT using immunohistochemistry [15–17], suggesting that immune checkpoint inhibitors in combination with CRT might enhance the response rate in advanced rectal cancer. Clinical trials are ongoing to test the effectiveness of the combination of conventional CRT and immune checkpoint inhibitors in rectal cancer (NCT 03127007, NCT03102047, NCT02948348). However, there were no reports to date examining the levels of sPD-L1 and soluble PD-1 (sPD-1) in rectal cancer patients before and after CRT.

The aim of the present study was to evaluate the changes in sPD-L1 and sPD-1 levels in patients with advanced rectal cancer treated with CRT, and the potential clinical implications associated with their expression.

## Materials and methods

### Patients

Serum samples before the start of CRT and after the completion of CRT (just before curative surgery) were prospectively collected from 117 patients with low rectal cancer treated with conventional long-course neoadjuvant CRT and who underwent surgery between July 2013 and April 2017 in our institution. Pretreatment clinical stage was assessed based on CT and MRI. The indications for CRT were: low rectal cancer with the inferior border located below the peritoneal reflection; clinical T3/T4 and/or node-positive disease by CT and/or MRI; and no evidence of distant metastases. CRT consisted of oral 5-fluorouracil and radiotherapy with a total dose of 50.4 Gy. Surgery was performed 6 to 8 weeks after the completion of CRT.

Clinical features of the selected patients were collected, including sex, age at surgery, distance of tumor from the anal verge, pretreatment carcinoembryonic antigen (CEA) levels, and clinical T and N categories. Surgical and pathological data collected included operative procedures, pathological T and N categories, tumor regression grade (TRG), histological type, lymphovascular invasion status, and adjuvant chemotherapy. The current retrospective study was approved by the institutional review board of the Cancer Institute Hospital in Tokyo, Japan (approval number “2013–1003”) and was conducted in compliance with the Declaration of Helsinki. Signed informed consent was obtained from all patients.

### sPD-L1 and sPD-1 ELISA

Blood samples were obtained from each patient during routine venipuncture. Whole blood was separated into serum and cellular fractions by centrifugation (3000 rpm for 10 min at 4°C). The supernatant serum was then aliquoted and stored at −80°C. Soluble PD-L1 and PD-1 levels were measured using a commercially available sandwich ELISA kit (Cloud-Clone Corp, Houston, TX, USA, catalog no. SEA788Hu (sPD-L1), SEA751Hu (sPD-1)) according to the manufacturer’s recommendations. Each sample was analyzed in duplicate. The minimum detectable concentration of sPD-L1 and sPD-1 was 0.057 ng/ml, and the detection range was 0.156 to 10 ng/mL. If assay values were less than 0.057 ng/mL, 0.01 ng/mL was substituted. The sPD-L1 and sPD-1 concentrations were defined as high when equal to or above 0.156 ng/mL (sPD-L1 high or sPD-1 high) and low when less than 0.156 ng/mL (sPD-L1 low or sPD-1 low). The intra-assay and inter-assay coefficients of variation were below 20%. sPD-L1 or sPD-1 concentrations were calculated using a standard curve.

### Tumor regression grade

TRG of the primary tumor after preoperative CRT was evaluated on hematoxylin-eosin–stained samples, according to Dworak’s criteria [18]: TRG 0, no regression; TRG 1, dominant tumor mass with obvious fibrosis and/or vasculopathy; TRG 2, dominant fibrotic changes with few tumor cells or groups; TRG 3, very few tumor cells in the fibrotic tissue with or without mucus substance; and TRG 4, no viable tumor cells, only fibrotic cells.

### Immunohistochemistry

Formalin-fixed, paraffin-embedded specimens before and after CRT (biopsy and resected specimens) from patients who underwent surgery before December 2014 were immunostained with rabbit monoclonal anti-PDL1 antibody (dilution 1:1200, ab205921; Abcam, Cambridge, UK), as described previously [15]. Moderate to strong expression in more than 5% of stromal immune cells was considered to be PD-L1 positive [15].

### Statistical analysis

Differences in categorical variables were compared using the Chi-squared test or Fisher’s exact test. Continuous variables were analyzed with the Mann–Whitney U-test. The level of sPD-L1 or sPD-1 before and after CRT was compared using the Wilcoxon-matched pairs signed rank test. The Kaplan–Meier method with the log-rank test was used for survival analyses. DFS was defined as the time from surgery to any recurrence or death from any cause. All P values less than 0.05 were considered significant. Uni- and multivariate analyses were performed using the Cox proportional hazard model to evaluate the predictors of recurrence. Variables with P values less than 0.2 in the univariate analyses were examined by multivariate analysis. Statistical analysis was performed using GraphPad Prism 7 software (GraphPad Software Inc., San Diego, CA, USA) or JMP software V10.0.2 (SAS Institute Inc.; Cary, NC, USA).

## Results

Table 1 shows the clinicopathological characteristics of the 117 patients enrolled in the present study. The median distance of the tumor from the anal verge was 40 mm (range, 0 to 80 mm). Most of the patients were diagnosed as T3.

**Table 1:** Clinicopathological characteristics of the 117 patients.

Sex	
Male	77 (65.8%)
Female	40 (34.2%)
Age (years) (range)	61 (27–79)
Distance of tumor from AV (mm) (range)	40 (0–80)
Baseline CEA >5 ng/ml	35 (29.9%)
Clinical T category	
T2	1 (0.9%)
T3	105 (89.7%)
T4	11 (9.4%)
Clinical stage	
II	55 (47.0%)
III	62 (53.0%)
Histological type	
Well/moderate	113 (96.6%)
poor	4 (3.4%)
Operative procedure	
Sphincter preserving	81 (69.2%)
Sphincter non-preserving	36 (30.8%)
ypT category	
ypT0	12 (10.2%)
ypTis	2 (1.7%)
ypT1	10 (8.6%)
ypT2	40 (34.2%)
ypT3	51 (43.6%)
ypT4	2 (1.7%)
ypN category	
ypN0	92 (78.6%)
ypN1	19 (16.2%)
ypN2	6 (5.2%)
Tumor regression grade	
TRG1	37 (31.6%)
TRG2	63 (53.8%)
TRG3	4 (3.4%)
TRG4	13 (11.2%)
Adjuvant chemotherapy	42 (35.9%)

Data are n (%) or median (range). AV, anal verge. CEA, carcinoembryonic antigen. CRT, chemoradiotherapy. TRG, tumor regression grade.

**Fig 1:**
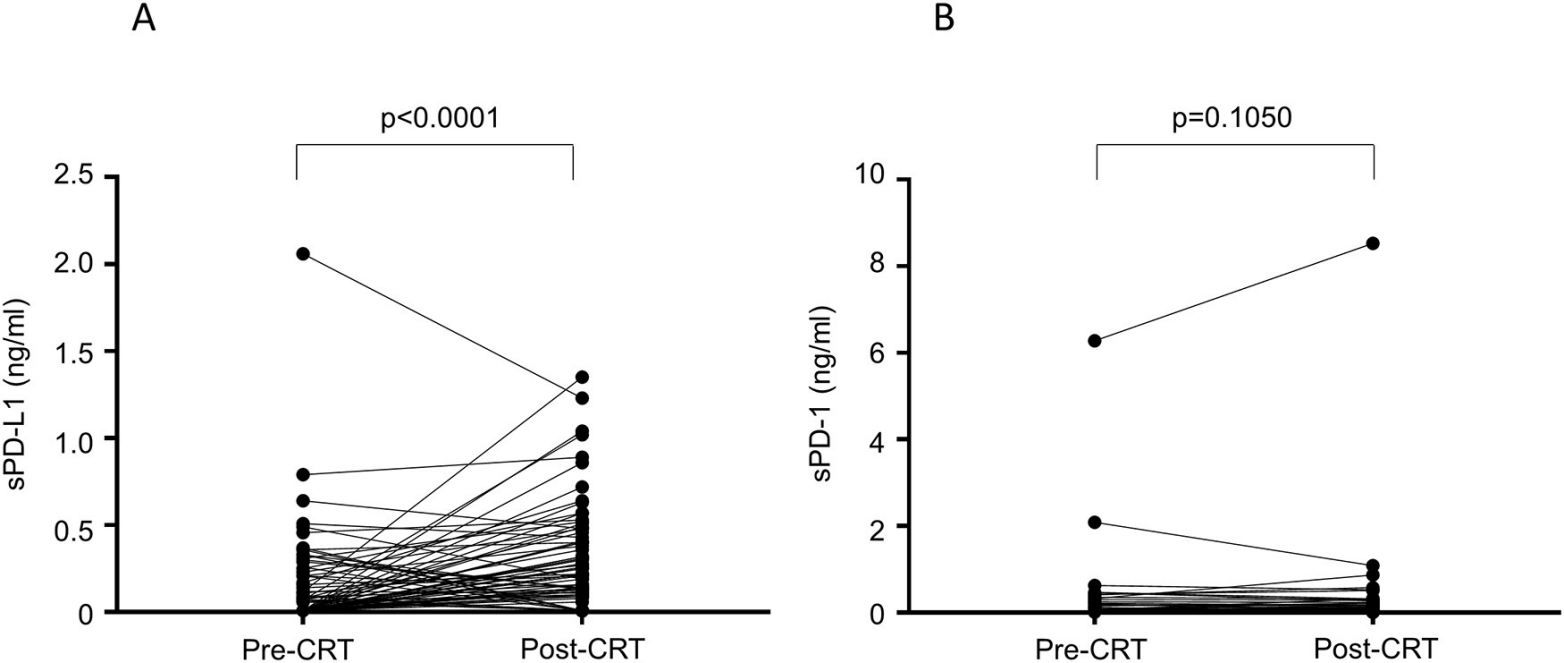
Changes in the soluble programmed cell death-ligand 1 (PD-L1) and soluble programmed cell death-1 (PD-1) expression levels before and after chemoradiotherapy. (A) Change in the sPD-L1 level. (B) Change in the sPD-1 level.

The serum concentrations of sPD-L1 and sPD-1 were examined pre- and post-CRT. sPD-L1 was evaluated in all (117) patients before CRT and in 91 patients after CRT. The level of sPD-L1 was below the lower limit of detection in 67 patients (57%; n = 117) before CRT and 27 patients (30%; n = 91) after CRT. sPD-L1 level significantly increased after CRT (p < 0.0001) (Fig 1A). sPD-1 was evaluated in 113 patients before CRT and 88 patients after CRT. The level of sPD-1 was below the lower limit of detection in 80 patients (71%; n = 113) before CRT and 53 patients (60%; n = 88) after CRT. The level of sPD-1 was not significantly different between pre- and post-CRT (p = 0.1050; Fig 1B).

Table 2 shows the patients’ characteristics according to serum sPD-L1 levels (high or low) pre- or post-CRT. High sPD-L1 before CRT was significantly associated with younger age (p = 0.044), and high sPD-L1 after CRT was significantly associated with lymphovascular invasion (p = 0.021). High sPD-1 before CRT and after CRT were significantly associated with a longer distance of the tumor from the anal verge (both p < 0.001) and higher rates of sphincter-preserving surgery (p = 0.007 and p = 0.004, respectively; S1 Table).

**Table 2:** Patient characteristics according to serum sPD-L1 levels pre- or post-chemoradiotherapy.

	Pre-CRT (N = 117)			Post CRT (N = 91)		
	sPD-L1 low (N = 87)	sPD-L1 high (N = 30)	*P*	sPD-L1 low (N = 45)	sPD-L1 high (N = 46)	*P*
Sex			0.436			0.274
Male	59 (67.8%)	18 (60.0%)		27 (60.0%)	33 (71.7%)	
Female	28 (32.2%)	12 (40.0%)		18 (40.0%)	13 (28.3%)	
Age (years) (range)	63 (27–79)	57 (29–75)	0.044	63 (34–77)	61 (27–79)	0.744
Distance of tumor from AV (mm) (range)	40 (0–80)	35 (0–70)	0.096	40 (0–70)	40 (0–80)	0.745
Pretreatment CEA (ng/mL) (range)	3.5 (0.5–104.1)	3.4 (0.9–12.6)	0.195	3.4 (1.2–58.5)	3.4 (0.5–104.1)	0.411
Operative procedure			0.591			0.207
Low anterior resection	36 (41.4%)	10 (33.3%)		21 (46.7%)	14 (30.4%)	
Intersphincteric resection	26 (29.9%)	8 (26.7%)		11 (24.4%)	18 (39.1%)	
Hartmann’s procedure	1 (1.1%)	0 (0%)		1 (2.2%)	0 (0%)	
Abdominoperineal resection	24 (27.6%)	12 (40.0%)		12 (26.7%)	14 (30.4%)	
Clinical T category			0.247			0.573
T2	1 (1.1%)	0 (%)		1 (2.2%)	0 (0%)	
T3	80 (92.0%)	25 (83.3%)		40 (88.9%)	41 (89.1%)	
T4	6 (6.9%)	5 (26.7%)		4 (8.9%)	5 (10.9%)	
Clinical stage			0.372			0.114
II	43 (49.4%)	12 (40.0%)		27 (60.0%)	20 (43.5%)	
III	44 (50.6%)	18 (60.0%)		18 (40.0%)	26 (56.5%)	
ypT category			0.408			0.312
ypT0	10 (11.5%	2 (6.7%)		6 (13.3%)	4 (8.7%)	
ypTis	2 (2.2%)	0 (0%)		2 (4.4%)	0 (0%)	
ypT1	9 (10.3%)	1 (3.3%)		2 (4.4%)	3 (6.5%)	
ypT2	26 (29.9%)	14 (46.7%)		19 (42.2%)	14 (30.4%)	
ypT3	39 (45.0%)	12 (40.0%)		16 (35.6%)	23 (50.0%)	
ypT4	1 (1.1%)	1 (3.3%)		0 (0%)	2 (4.4%)	
ypN category			0.731			0.410
ypN0	69 (79.3%)	23 (76.7%)		38 (84.4%)	36 (78.3%)	
ypN1	13 (14.9%)	6 (20.0%)		6 (13.3%)	10 (22.7%)	
ypN2	5 (5.8%)	1 (3.3%)		1 (2.2%)	0 (0%)	
Tumor regression grade			0.490			0.299
TRG1	27 (31.0%)	10 (33.3%)		11 (24.4%)	18 (39.1%)	
TRG2	45 (51.7%)	18 (60.0%)		27 (60.0%)	20 (43.5%)	
TRG3	4 (4.6%)	0 (0%)		1 (2.2%)	3 (6.5%)	
TRG4	11 (12.7%)	2 (6.7%)		6 (13.3%)	5 (10.9%)	
Histological type			1.000			1.000
Well/moderate	84 (96.5%)	29 (96.7%)		44 (97.8%)	45 (97.8%)	
Poor	3 (3.5%)	1 (3.3%)		1 (2.2%)	1 (2.2%)	
Lymphovascular invasion			0.077			0.021
Negative	51 (58.6%)	12 (40.0%)		30 (66.7%)	19 (41.3%)	
Positive	36 (41.4%)	18 (60.0%)		15 (33.3%)	27 (58.7%)	
Adjuvant chemotherapy			0.434			0.824
No	54 (62.1%)	21 (70.0%)		31 (68.9%)	30 (65.2%)	
Yes	33 (37.9%)	9 (30.0%)		14 (31.1%)	16 (44.8%)	

Data are n (%) or median (range). AV, anal verge. CEA, carcinoembryonic antigen. CRT, chemoradiotherapy. TRG, tumor regression grade.

Next, we evaluated the correlation between sPD-L1 level and tissue PD-L1 expression on stromal immune cells by immunohistochemistry. Among the 44 patients for whom we could evaluate both PD-L1 expression in biopsy specimens and sPD-L1 level before CRT, the percentage of high sPD-L1 level was not significantly different between high and low PD-L1 expression in biopsy specimens [5.6% (1/18) vs 15.4% (4/26); p = 0.6337]. Among the 33 patients for whom we could evaluate both PD-L1 expression in resected specimens and sPD-L1 level after CRT, the percentage of high sPD-L1 level was not significantly different between high and low PD-L1 expression in resected specimens [61.1% (11/18) vs 46.7% (7/15); p = 0.4939].

**Fig 2:**
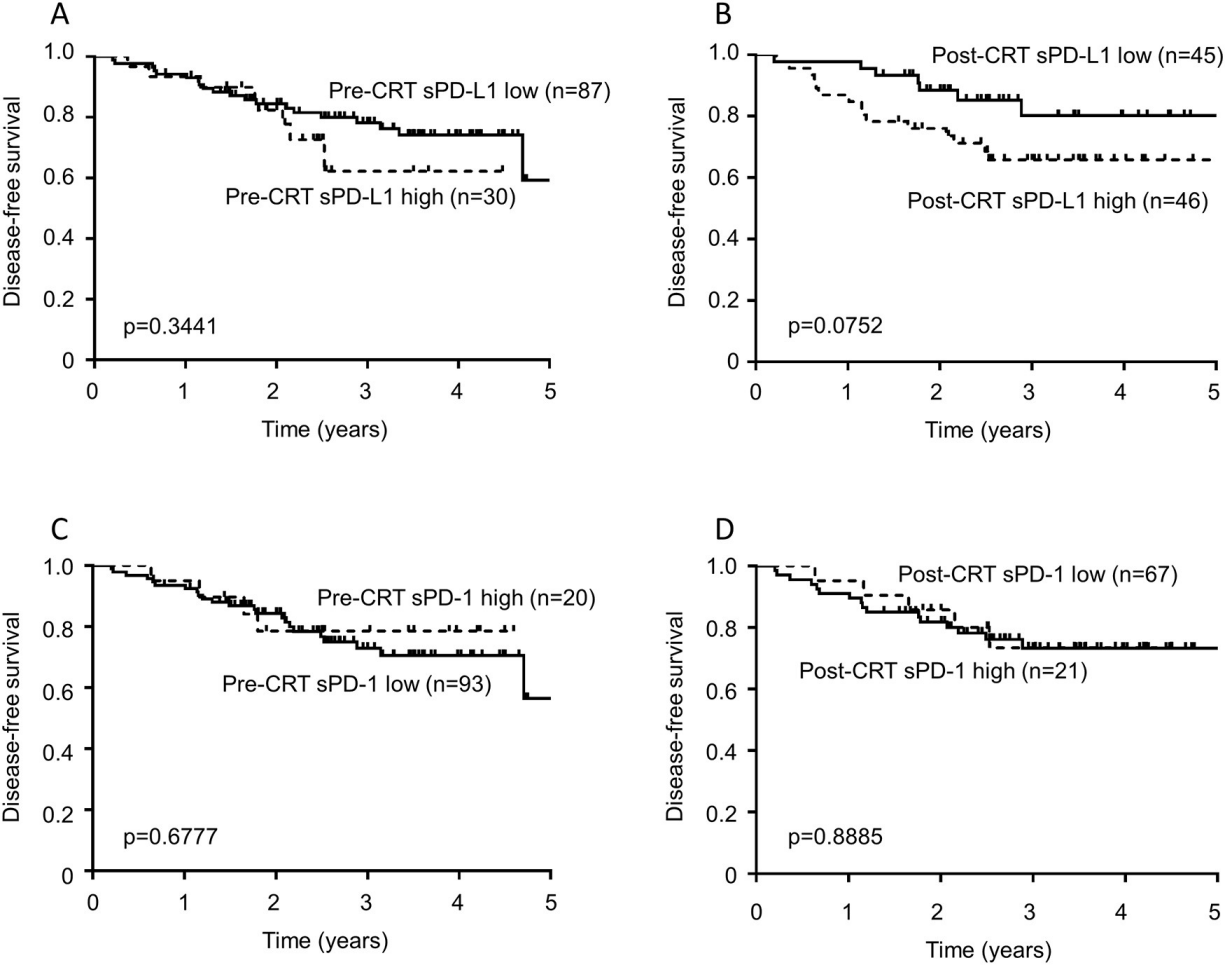
Disease-free survival (DFS) associated with the levels of soluble programmed cell death-ligand 1 (PD-L1) and soluble programmed cell death-1 (PD-1) before or after chemoradiotherapy (CRT). (A) DFS associated with sPD-L1 level before CRT. (B) DFS associated with sPD-L1 level after CRT. (C) DFS associated with sPD-1 level before CRT. (D) DFS associated with sPD-1 level after CRT.

The median follow-up duration was 33.7 months (8.0 to 60.6 months). Patients with a high sPD-L1 level after CRT showed a trend for worse disease-free survival (DFS) than those with a low sPD-L1 level (p = 0.0752); although, the p-value did not reach statistical significance (Fig 2B). The sPD-L1 level before CRT was not associated with DFS (p = 0.3441; Fig 2A). The concentrations of sPD-1 both pre- and post-CRT were not associated with DFS (p = 0.6777 and p = 0.8885, respectively; Fig 2C and 2D). Table 3 shows the results of the univariate and multivariate analyses of factors associated with DFS. In the univariate analysis, male sex (p = 0.0490), pathological T3/4 (p = 0.0008), pathological node metastasis (p < 0.0001), and the presence of lymphovascular invasion (p = 0.0005) were significantly associated with worse DFS. In the multivariate analysis, pathological node metastasis remained an independent predictor of DFS (HR: 4.61, 95% CI: 1.82 to 12.32, p = 0.0013). The post-CRT sPD-L1 level did not reach statistical significance (HR: 1.85, 95% CI: 0.77 to 4.89, p = 0.1727).

**Table 3:** Univariate and multivariate analysis of factors associated with disease-free survival.

Variable	Univariate analysis		Multivariate analysis	
	HR (95% CI)	p value	HR (95% CI)	p value
Age (<70 y vs. ≥70 y)	0.85 (0.25–2.28)	0.7679		
Sex (female vs male)	2.70 (1.00–9.35)	0.0490	2.28 (0.83–7.99)	0.1149
Distance from the AV (≤40 mm vs >40 mm)	0.57 (0.22–1.36)	0.2125		
Baseline CEA >5 (no vs yes)	1.99 (0.82–4.64)	0.1245	1.23 (0.48–3.00)	0.6636
pathological T category (others vs 3/4)	4.67 (1.84–14.21)	0.0008	2.34 (0.85–7.62)	0.1046
Pathological nodal metastasis (no vs yes)	7.86 (3.37–19.19)	<0.0001	4.61 (1.82–12.32)	0.0013
Lymphovascular invasion (no vs yes)	4.93 (1.95–15.02)	0.0005	1.96 (0.69–6.55)	0.2163
sPD-L1 level after CRT (low vs high)	2.21 (0.93–5.80)	0.0721	1.85 (0.77–4.89)	0.1727

AV, anal verge. CEA, carcinoembryonic antigen. CRT, chemoradiotherapy

## Discussion

In the present study, we showed that sPD-L1, but not sPD-1, significantly increased after CRT. Patients with high sPD-L1 levels after CRT showed a trend toward worse DFS than those with low sPD-L1 levels. This is the first study to demonstrate the change in sPD-L1 levels following CRT in patients with advanced rectal cancer.

Previous studies have shown that sPD-L1 is elevated in several malignancies [7, 9], with cut-off values ranging from 0.00965 to 7.32 ng/mL, and positivity rate from 29.8% to 56.0% [9]. In the present study, we determined the cut-off value based on the median concentration of sPD-L1 after CRT (0.16 ng/mL). We showed that sPD-L1 and sPD-1 levels before and after CRT were not significantly associated with pathological response to CRT, such as ypT, ypN, and TRG, and suggest that neither sPD-L1 nor sPD-1 is useful to predict pathological response to CRT in rectal cancer.

Although sPD-L1 has not been determined in rectal cancer before, previous studies have reported membrane-bound PD-L1 and PD-1 expression by immunohistochemistry in colorectal cancer. Lee et al. showed that PD-L1 positivity in tumor cells and PD-1 positivity in tumor-infiltrating lymphocytes in mismatch-repair proficient colorectal cancer were only 2% and 13%, respectively, which are significantly lower than the positivity rates (18% and 50%) reported for mismatch-repair deficient colorectal cancer [12]. Considering the low percentage of mismatch-repair deficient tumors in rectal cancer [19], it is speculated that sPD-L1 is likely produced by cells other than the tumor cells in rectal cancer.

In the present study, we showed that sPD-L1 levels significantly increased after CRT. Consistent with our results, a previous study of patients with hepatocellular carcinoma who were treated with radiotherapy showed significantly elevated levels of sPD-L1 after radiotherapy [20]. Increased levels of sPD-L1 after CRT suggests that PD-L1 could be a therapeutic target in combination with CRT in advanced rectal cancer. Using pre-treatment biopsy specimens and resected specimens from patients with rectal cancer who were treated with CRT, two studies showed using immunohistochemistry that PD-L1 expression on tumor cells significantly increased after CRT [16, 17]. We have also shown that PD-L1 expression on stromal immune cells significantly increased after CRT in advanced rectal cancer [15]. However, two conflicting studies show no significant change in tumoral PD-L1 expression after CRT [15, 21], and, in the present study, we could not detect a significant association between sPD-L1 level and local PD-L1 expression on stromal immune cells by immunohistochemistry. A similar absence of an association between sPD-L1 and PD-L1 expression on tumor cells or on non-malignant cells in the tumor microenvironment was reported in diffuse large B-cell lymphoma [22, 23] and pancreatic cancer [24]. Therefore, it is speculated that the mechanism of sPD-L1 production is complex and not directly related to PD-L1 expression at the tumor site. Furthermore, there might be multiple sources of sPD-L1. Indeed, one previous study suggested that myeloid-derived cells may be a natural source of sPD-L1 [25]. With regard to sPD-1 levels, although sPD-1 levels are reported to increase during chemotherapy in non-small cell lung cancer [26], we found no significant increase in sPD-1 after CRT.

In our study, we found that high sPD-L1 levels after CRT tended to be associated with worse DFS. However, in the multivariate analysis, we found that pathological nodal metastasis was the only independent predictor of worse DFS, and we failed to show an independent association of sPD-L1 level with DFS. Although this might be explained by the small sample size—and further studies with a larger number of patients are necessary to clarify the relationship between sPD-L1 level and DFS—our data suggest that sPD-L1 level might not be strongly associated with DFS compared to ypN status. However, the trend toward a worse DFS suggests that high sPD-L1 levels after CRT might reflect the systemic immunosuppressive state, and the combination of CRT and immune checkpoint inhibitors might be effective to overcome the immunosuppressive environment in these patients. Recently, a preliminary report from a phase Ib/II trial suggested that the combination of CRT and anti-PD-1 antibody (nivolumab) might improve the complete response rate in advanced rectal cancer [27].

There are several limitations in our study. First, this study was a single-center, retrospective, exploratory analysis, with a small sample size. Second, the evaluation of sPD-L1 and sPD-1 using an enzyme-linked immunosorbent assay (ELISA) kit is not yet standardized, and the optimal cut-off values have yet to be established. Third, the follow-up period was not long enough. Finally, the blood sampling was limited to before and after CRT, and we could not determine the change in sPD-L1 level after surgery.

In conclusion, in patients with advanced rectal cancer, sPD-L1 levels significantly increased after CRT, and patients with high sPD-L1 levels after CRT tended to show worse DFS. The increased levels of sPD-L1 after CRT suggests that the combination of CRT and immune checkpoint inhibitors may be a promising therapeutic strategy for patients with advanced rectal cancer.

## Supporting information

S1 Table Patient characteristics according to serum sPD-1 levels pre- or post- chemoradiotherapy. (DOCX)
