# Supplementary material for: Clinical significance of soluble programmed cell death-1 and soluble programmed cell death-ligand 1 in patients with locally advanced rectal cancer treated with neoadjuvant chemoradiotherapy
Source: PLoS One. 2019 Feb 26;14(2):e0212978. doi: 10.1371/journal.pone.0212978 (PMC6390997; doi:10.1371/journal.pone.0212978)
Supplement: S1 Table — (DOCX) [file pone.0212978.s001.docx]

Supplementary Table 1 Patient characteristics according to serum sPD-1 levels pre- or post- chemoradiotherapy

|  | Pre-CRT  (N=113) | |  |  | Post CRT  (N=88) | |  |
| --- | --- | --- | --- | --- | --- | --- | --- |
|  | sPD-1 low  (N=80) | sPD-1 high  (N=33) | *P* |  | sPD-1 low  (N=67) | sPD-1 high  (N=21) | *p* |
| Sex |  |  | 0.768 |  |  |  | 1.000 |
| Male | 51 (63.7%) | 22 (66.7%) |  |  | 45 (67.1%) | 14 (66.7%) |  |
| Female | 29 (36.3%) | 11 (33.3%) |  |  | 22 (32.9%) | 7 (33.3%) |  |
| Age (years) (range) | 61 (27–78) | 60 (36–79) | 0.678 |  | 61 (27–78) | 63 (36–79) | 0.360 |
| Distance of tumor from  AV (mm) (range) | 40 (0–75) | 50 (10–80) | <0.001 |  | 35 (0–75) | 55 (30–80) | <0.001 |
| Pretreatment CEA  (ng/mL) (range) | 3.6 (0.5–58.5) | 3.2 (0.9–104.1) | 0.695 |  | 3.4 (0.5–58.5) | 3.4 (0.9–104.1) | 0.344 |
| Pretreatment CA19-9  (ng/mL) (range) | 7.1 (2–434.8) | 5.9 (2–80.6) | 0.508 |  | 7.0 (2–434.8) | 6.9 (2–135.5) | 0.829 |
| Operative procedure  Low anterior resection  Intersphincteric resection  Hartmann’s procedure  Abdominoperineal resection | 23 (28.7%)  28 (35.0%)  1 (1.3%)  28 (35.0%) | 21(63.6%)  6 (18.2%)  0 (0%)  6 (18.2%) | 0.007 |  | 19 (28.4%)  25 (37.3%)  1 (1.5%)  22 (32.8%) | 15 (71.4%)  3 (14.3%)  0 (0%)  3 (14.3%) | 0.004 |
| Clinical T category  T2  T3  T4 | 1 (1.2%)  71 (88.8%)  8 (10.0%) | 0 (0%)  30 (90.9%)  3 (9.1%) | 0.801 |  | 1 (1.5%)  61 (91.0%)  5 (7.5%) | 0 (0%)  18 (85.7%)  3 (14.3%) | 0.554 |
| Clinical stage  II  III | 37 (46.3%)  43 (53.7%) | 16 (48.5%)  17 (51.5%) | 0.829 |  | 37 (55.2%)  30 (44.8%) | 9 (42.9%)  12 (57.1%) | 0.322 |
| ypT category  CR  ypTis  ypT1  ypT2  ypT3  ypT4 | 7 (8.7%)  2 (2.5%)  9 (11.2%)  27 (33.8%)  35 (43.8%)  0 (0%) | 4 (12.1%)  0 (0%)  1 (3.0%)  10 (30.3%)  16 (48.5%)  2 (6.1%) | 0.160 |  | 7 (10.4%)  0 (0%)  5 (7.5%)  28 (41.8%)  27 (40.3%)  0 (0%) | 3 (14.3%)  0 (0%)  0 (0%)  5 (23.8%)  11 (52.4%)  2 (9.5%) | 0.059 |
| ypN category  ypN0  ypN1  ypN2 | 62 (77.5%)  14 (17.5%)  4 (5.0%) | 27 (81.8%)  4 (12.1%)  3 (9.1%) | 0.583 |  | 57 (85.1%)  10 (14.9%)  0 (0%) | 16 (76.2%)  5 (23.8%)  0 (0%) | 0.338 |
| Tumor regression grade  1  2  3  4 | 26 (32.5%)  44 (55.0%)  2 (2.5%)  8 (10.0%) | 9 (27.3%)  19 (57.6%)  1 (3.0%)  4 (12.1%) | 0.948 |  | 20 (29.9%)  37 (55.2%)  2 (3.0%)  8 (11.9%) | 8 (38.1%)  8 (38.1%)  2 (9.5%)  3 (14.3%) | 0.348 |
| Histological type  Well/mod  por | 77 (96.3%)  3 (3.7%) | 32 (97.0%)  1 (3.0%) | 1.000 |  | 65 (97.0%)  2 (3.0%) | 21 (100%)  0 (0%) | 1.000 |
| Lymphovascular invasion  Negative  Positive | 45 (56.3%)  35 (43.7%) | 17 (51.5%)  16 (48.5%) | 0.645 |  | 36 (53.7%)  31 (46.3%) | 11 (52.4%)  10 (47.6%) | 1.000 |
| Adjuvant chemotherapy  No  Yes | 48 (60.0%)  32 (40.0%) | 24 (72.7%)  9 (27.3%) | 0.200 |  | 45 (67.2%)  22 (32.8%) | 15 (71.4%)  6 (18.6%) | 0.793 |

Data are n (%) or median (range). AV, anal verge. CEA, carcinoembryonic antigen. CRT, chemoradiotherapy. TRG, tumor regression grade.
